# Minimally Invasive Tissue Sampling Surveillance Alliance—Facilitating the Expansion of Pathology-Based Mortality Surveillance

**DOI:** 10.1093/cid/ciab827

**Published:** 2021-12-15

**Authors:** Norman J Goco, Elizabeth M McClure, Natalia Rakislova, Quique Bassat

**Affiliations:** 1 RTI International, Research Triangle Park, North Carolina, USA; 2 ISGlobal, Hospital Clínic, Universitat de Barcelona, Barcelona, Spain; 3 Department of Pathology, Hospital Clínic, Universitat de Barcelona, Barcelona, Spain; 4 Centro de Investigação em Saúde de Manhiça, Maputo, Mozambique; 5 Institució Catalana de recerca i estudis avançats (ICREA), Pg. Lluís Companys 23, 08010 Barcelona, Spain; 6 Pediatrics Department, Hospital Sant Joan de Déu, Universitat de Barcelona, Esplugues, Barcelona, Spain; 7 Consorcio de Investigación Biomédica en Red de Epidemiología y Salud Pública, Madrid, Spain

**Keywords:** pathology-based, mortality surveillance, cause of death

## Abstract

The Minimally Invasive Tissue Sampling (MITS) Surveillance Alliance was created with funding from the Bill & Melinda Gates Foundation to expand pathology-based mortality surveillance and to support the generation of improved cause-of-death (CoD) data. MITS, also known as minimally invasive autopsy, has evolved to become an important tool to improve CoD ascertainment. Here, we describe the 18 articles included in this supplement that present advanced methods for improving MITS and related areas of research, summarize the expansion of the use of MITS, report on findings from a variety of research projects, and address the importance of postmortem approaches taken during the novel coronavirus disease 2019 pandemic. Support by the scientific and global health community for enhancements and innovation is needed for the broader adoption of MITS-informed CoD as a critical tool to better understand mortality in low- and middle-income countries and identify interventions for the prevention of premature death.

## COMMENTARY

The coronavirus disease 2019 (COVID-19) pandemic has highlighted, again, the importance of accurate cause-of-death (CoD) information when developing effective responses to reduce mortality. Postmortem examination is an important component to understand why a person dies. Unfortunately, autopsy rates have fallen over the last few decades globally and remain infrequently conducted in low- and middle-income countries (LMICs) where resources are scarce and the acceptability of autopsy procedures varies [1]. Within the last decade, there has been a renewed interest in postmortem studies to improve CoD attribution, particularly for mortality surveillance where civil registration and vital statistics systems are not reliable or nonexistent. Minimally invasive tissue sampling (MITS), also known as minimally invasive autopsy, has evolved to become an important tool to improve CoD ascertainment, particularly where complete diagnostic autopsies are not routinely conducted or challenging to perform [2, 3]. Validation studies have demonstrated good performance and high concordance with complete diagnostic autopsy across populations, particularly when infectious diseases are an important contributor to the immediate or underlying causes of death [4–7].

The MITS Surveillance Alliance was formed in 2018 with funding from the Bill & Melinda Gates Foundation to expand pathology-based mortality surveillance and support the generation of improved CoD data.

In this supplement, we have included a set of articles that collectively show the catalytic work that the MITS Alliance has fostered in the last 3 years. The articles present advanced methods for improving MITS and related areas of research, summarize the expansion of the use of MITS through capacity-building and sharing lessons learned, report on findings from a variety of research projects, and address the importance of postmortem approaches in the novel COVID-19 pandemic.

## ARTICLES ON METHODOLOGY

An important step in improving the accuracy and quality of CoD data is the standardization of MITS procedures, training, and CoD attribution based on MITS findings. For this purpose, the MITS Alliance brought together experts from ISGlobal, the US Centers for Disease Control and Prevention Infectious Disease Pathology Branch, and the University of Nairobi Department of Pathology. This collaboration has been fundamental to the development of a training center based in Kenya [8]. Researchers from projects in India, Kenya, Pakistan, and Bangladesh came together to share best practices in approaching community sensitization and the consent process in preparation for implementing MITS in populations and cultures where postmortem studies are challenging to conduct because of specific religious practices surrounding the phenomenon of death and the process of burial and grief [9].

In this series of articles, we share advances for improving the MITS methodology and related areas of research important to CoD determination, including challenges of interpreting the contribution of multiple factors when using multiplex molecular testing. Letang et al explored CoD among people living with human immunodeficiency virus (PLHIV) in populations of Brazil and Mozambique using MITS and comparing their performance with the results of complete autopsy. MITS was found to accurately identify the microorganisms that contribute to the CoD in 89% of the cases and could easily replace the complete autopsy, become a useful tool in monitoring the CoD, and highlight gaps in the management of opportunistic infections among PLHIV [10].

As part of many MITS protocols, multiplex molecular testing using the TaqMan® array card (TAC) is used for the identification of pathogens that contribute to the causal pathway in the determination of CoD. Two analyses from a study conducted at the Kenyatta National Hospital in Nairobi examine the performance and role of TAC compared with other diagnostic testing in identifying pathogens, including the assessment of and the postmortem interval on pathogen detection using TAC within a MITS protocol. Ritter et al evaluated MITS results among 20 deceased children using TAC, histopathology, special stains, immunohistochemistry, and molecular testing using polymerase chain reaction on formalin-fixed, paraffin-embedded tissues. The authors noted that agreement across these testing methods varied and, thus, emphasized the need for careful interpretation of TAC results in the context of the clinical and histopathologic findings, along with other diagnostic testing, for CoD determination [11]. In the second study, Dawa et al determined that reliable microbiological results may be obtained for up to 96 hours postmortem if adequate body storage and aseptic conditions in specimen collection can be maintained. However, as inconsistent pathogen detection was reported across sample batches, their results also emphasize the need for careful interpretation in combination with other diagnostic information for proper CoD determination [12].

Hwang et al share a streamlined approach to facilitate expert panel evaluation, an important step in the understanding and attribution of CoD, as part of the Project to Understand and Research Preterm Pregnancy Outcome in South Asia (PURPOSe). This innovation increased the efficiency of the expert panel review process and also limited the introduction of bias into the evaluation [13].

Two articles included in this supplement specifically address undernutrition as a CoD among children in LMICs. Paganelli et al bring together several partners and external stakeholders with expertise in public health, nutrition, and infectious diseases to develop guidelines for the attribution of undernutrition diagnosis as a CoD in children aged <5 years. The standards were developed to provide a systematic approach for understanding, assigning, and coding undernutrition as a CoD [14]. Fuetz et al present their evaluation of the feasibility of innovative MITS including intestinal sampling while also studying whether autolysis precludes the utility of enteric biopsies and to determine the histopathology findings among deceased children in Malawi with acute illness or undernutrition. This innovative approach shows promise, as their study demonstrated that intestinal sampling was feasible with greater than 90% success in obtaining targeted tissue; they were able to visualize histopathological changes that can inform CoD [15].

## CAPACITY BUILDING AND LESSONS LEARNED

This supplement highlights initial findings from 19 projects funded through the MITS Alliance small grants program, which is supporting MITS-informed CoD research across 3 continents (Africa, Asia, and Latin America) in 12 countries (Argentina, Brazil, Ethiopia, Kenya, Nigeria, Rwanda, Tanzania, Zambia, India, Kazakhstan, Nepal, and Pakistan) [16].

Contributing information valuable for the expansion of MITS as a tool for improved CoD attribution, 3 articles focus on building capacity, lessons learned from small-scale implementation projects, and an evaluation of the cost of implementing MITS. Paganelli et al describe the process undertaken to establish a training center for MITS at the Kenyatta National Hospital in Nairobi, including the adaptation and development of standard operating procedures and training materials [8]. Subedi et al share the experience at sites in Nepal, Rwanda, and Tanzania in implementing small-scale MITS projects, describing the process and lessons learned from each step of training, site assessment findings, and the use of expert support for improving quality and procedures. Many of the challenges encountered were common, and their experiences are informative for future adopters of MITS in similar circumstances [17].

Morrison et al present results from a cost evaluation of MITS conducted among small studies that were naive to the use of this technique. The estimates presented may serve as guidance for policymakers interested in implementing MITS-informed CoD attribution to support mortality surveillance or special studies [18].

## FINDINGS: MITS SURVEILLANCE ALLIANCE GRANTEE PROJECTS AND PARTNERS

Matthew et al present results from a pilot study to explore the use of MITS to diagnose CoD among neonates with neurological insults in a neonatal intensive care unit in India. The pilot study demonstrated that MITS allowed for an accurate and adequate diagnosis for determining CoD due to neurological insults, particularly in settings where consent for complete autopsy may be difficult to obtain [19].

Suraj et al studied the burden of antimicrobial resistance with their postmortem investigation of 100 cases using MITS in Nepal. Their study highlighted the importance of robust antimicrobial resistance surveillance and improved infection prevention control, as an increased risk of multidrug-resistant organisms was found among hospital deaths [20].

In this collection, we also include findings from other MITS Alliance partners. In Sunder et al, PURPOSe investigators present a substudy examining the acceptability of MITS in women who experienced a stillbirth or preterm live birth with a fatal outcome. Cultural issues and the potential delay of funeral practices were the primary reasons for refusal of MITS by families studied in India and Pakistan, while the parents’ interest in understanding the CoD was the primary reason for accepting the MITS procedure [21]. Guruprasad et al share observations using MITS to examine the lungs among 453 stillbirths and 352 neonatal deaths in tertiary-level facilities in India and Pakistan, concluding that MITS provided a reasonable alternative to complete autopsy to inform CoD [22].

From the Argentina MITS project, Caballero et al conducted a population-based postmortem study, incorporating MITS, verbal autopsy, and molecular analyses of samples, of community-based deaths among children aged <5 years within 6 poor and disadvantaged districts of the Province of Buenos Aires. They found that among 96 cases enrolled, the most common CoD was lung infection, with undernutrition being the most frequent comorbid condition [23].

## MITs STUDIES ON COVID-19

As with other global research activities, the COVID-19 pandemic has had a significant impact on the MITS Alliance community, particularly with the delay in implementation of research activities that were impacted by local restrictions on nonessential activities. The pandemic has presented an opportunity to support the response to this public health emergency through postmortem studies, sharing procedures for enhanced biosafety to enable researchers to move forward during a time of uncertainty and demonstrating the utility of MITS as an important tool to contribute to the understanding of a novel and very lethal disease [24–26]. In this collection, 4 articles focus on the use of MITS for postmortem study of COVID-19, addressing biosafety and its utility vs complete autopsy. Neto et al and Rakislova et al evaluated MITS in postmortem studies of COVID-19, with the former incorporating the ultrasound-guided approach among a population in Sao Paulo, Brazil, and the latter evaluating diagnostic performance of MITS compared with complete diagnostic autopsy in a population of the Barcelona metropolitan area, Spain. Both studies demonstrated the safety and utility of MITS for the identification and characterization of COVID-19 and potentially for use in both low- and high-resource settings [27, 28]. In Zambia, as part of the research response to the COVID-19 outbreak, Mudenda et al pivoted their existing postmortem research to examine the pathophysiology of COVID-19 through pathological changes, one of few postmortem studies of COVID-19 patients conducted in an LMIC [29]. Finally, Bassat et al makes a strong case for the use of MITS for the investigation of outbreaks and epidemics, as an alternative to complete diagnostic autopsy, particularly for infectious diseases of high lethality [30].

The MITS Surveillance Alliance aims to continue to incorporate findings from this community of practice to improve methods and streamline procedures to facilitate implementation and continue contributing to the Bill & Melinda Gates Foundation’s commitment to understanding death [31]. For example, an important objective to help improve efficiency and cost-effectiveness being addressed is the identification of the most important tissue and fluid samples that significantly contribute to the determination of CoD. Our partners at the Country-wide Mortality Surveillance for Action Mozambique Program have taken additional steps to use MITS-informed CoD data to refine, or “calibrate,” verbal autopsy data (the most widely available method used for CoD attribution in LMICs but unspecific and with suboptimal performance at the individual level) to improve the precision of population-based estimates of disease [32]. This approach has shown promise as part of an interim strategy to improve mortality surveillance until reliable data from mature civil registry and vital statistics systems are established.

The MITS Surveillance Alliance leaves a living legacy of several well-established MITS projects running at a global level. Its catalytic and multiplicative effect will transcend the duration of the grant and has facilitated the introduction of more reliable methods for CoD investigation, following standard and well-protocolized recommendations. The scientific community and global health researchers now need to continue to explore this and other innovations and to further facilitate broader adoption of MITS-informed CoD as a critical tool to better understand mortality in LMICs and identify interventions for the prevention of premature death.

## Notes

***Financial support.*** This work was supported by the MITS Surveillance Alliance. This work was supported, in whole or in part, by the Bill & Melinda Gates Foundation [OPP1180554]. Under the grant conditions of the Foundation, a Creative Commons Attribution 4.0 Generic License has already been assigned to the Author Accepted Manuscript version that might arise from this submission.

***Supplement sponsorship.*** This supplement is sponsored by MITS Surveillance Alliance Secretariat, led by RTI International, with funding from the Bill and Melinda Gates Foundation.

***Potential conflicts of interest.*** All authors: No reported conflicts of interest. All authors have submitted the ICMJE Form for Disclosure of Potential Conflicts of Interest. Conflicts that the editors consider relevant to the content of the manuscript have been disclosed.
